# An Unprecedented Efficiency with Approaching 21% Enabled by Additive-Assisted Layer-by-Layer Processing in Organic Solar Cells

**DOI:** 10.1007/s40820-024-01529-8

**Published:** 2024-10-14

**Authors:** Shuai Xu, Youdi Zhang, Yanna Sun, Pei Cheng, Zhaoyang Yao, Ning Li, Long Ye, Lijian Zuo, Ke Gao

**Affiliations:** 1 Key Laboratory of Advanced Green Functional Materials, College of Chemistry, Changchun Normal University, Changchun, 130032 People’s Republic of China; 2 Shandong Provincial Key Laboratory for Science of Material Creation and Energy Conversion, School of Chemistry and Chemical Engineering, Shandong University, Qingdao, 266237 People’s Republic of China; 3 College of Polymer Science and Engineering, Sichuan University, Chengdu, 610065 People’s Republic of China; 4 State Key Laboratory and Institute of Elemento-Organic Chemistry, Centre of Nanoscale Science and Technology and Key Laboratory of Functional Polymer Materials, Renewable Energy Conversion and Storage Center (RECAST), College of Chemistry, Nankai University, Tianjin, 300071 People’s Republic of China; 5 Institute of Polymer Optoelectronic Materials and Devices State Key Laboratory of Luminescent Materials and Devices, South China University of Technology, Guangzhou, 510640 People’s Republic of China; 6 Tianjin Key Laboratory of Molecular Optoelectronic Sciences, School of Materials Science and Engineering, Tianjin University, Tianjin, 300350 People’s Republic of China; 7 State Key Laboratory of Silicon Materials, MOE Key Laboratory of Macromolecular Synthesis and Functionalization, Department of Polymer Science and Engineering, Zhejiang University, Hangzhou, 310027 People’s Republic of China

**Keywords:** Organic solar cells, Additive-assisted layer-by-layer processing, Three-dimensional fibril morphology, Bulk *p-i-n *structure, Optical management

## Abstract

Recently published in Joule, Feng Liu and colleagues from Shanghai Jiaotong University reported a record-breaking 20.8% power conversion efficiency in organic solar cells (OSCs) with an interpenetrating fibril network active layer morphology, featuring a bulk *p-i-n* structure and proper vertical segregation achieved through additive-assisted layer-by-layer deposition. This optimized hierarchical gradient fibrillar morphology and optical management synergistically facilitates exciton diffusion, reduces recombination losses, and enhances light capture capability. This approach not only offers a solution to achieving high-efficiency devices but also demonstrates the potential for commercial applications of OSCs.

## Highlights

- Additive-assisted layer-by-layer (LBL) deposition enables organic solar cells to achieve an unprecedented power conversion efficiency of 20.8%, the highest efficiency to date.
- The gradient fibrillar morphology enabled by additive-assisted LBL processing promotes the formation of bulk *p-i-n* structure, improving exciton and carrier diffusion, and reducing recombination losses.
- The wrinkle pattern morphology achieved by additive-assisted LBL processing is constructed to enhance the light capture capability.

## Graphical abstract

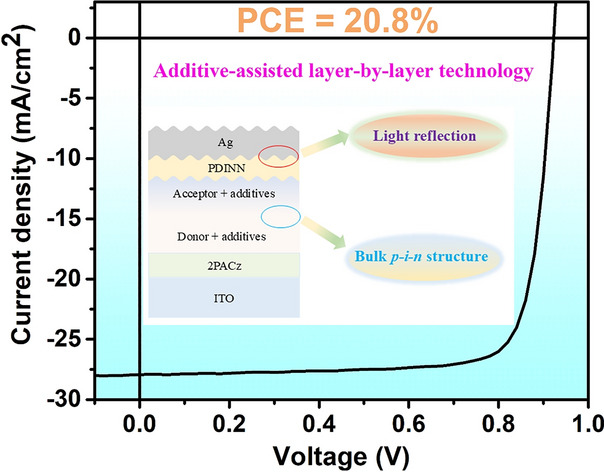

 Organic solar cells (OSCs) possess the potential for a variety of future applications, including flexible and semi-transparent installations, particularly in the integration with buildings for smart glass windows or device integration, highlighting their unique characteristics. Advancements in polymer donor and non-fullerene acceptor materials have infused the OSC field with renewed optimism [1–3]. These material innovations have significantly broadened the light spectrum absorption range of the active layers and reduced energy losses in devices, leading to a remarkable breakthrough with a power conversion efficiency (PCE) exceeding 19% [4]. In addition, device fabrication and optimization techniques with bulk heterojunction type (BHJ) and layer-by-layer type (LBL) have been greatly improved. The optimal device engineering mainly involves regulating crystallinity and phase separation length scales to improve key morphological parameters in the process of photon-to-electron conversion and transport. The self-assembly behavior of donor/acceptor materials provides a highly crystalline framework for carrier transport channels, reducing the phase size from exciton to carrier processing. However, in BHJ thin-film devices, donor/acceptor blended films can create larger domain areas and poor vertical phase separation, which significantly hinders carrier transport across the films. Compared with the BHJ processing, LBL processing is an effective strategy to address the aforementioned issues. The polymer donor layer at the bottom shows good crystalline connectivity, while the acceptor material partially swells on top, demonstrating a favorable gradient distribution morphology in the vertical direction and thus enabling high performance for OSCs.

Writing in Joule (https://doi.org/10.1016/j.joule.2024.08.001), Feng Liu's group [5] reported an unprecedented PCE of 20.8% for small-area devices (certified as 20.1%) and 17.0% for minimodule devices. These exciting results have set a new milestone for further improving the efficiency of OSCs and paved the path to the commercialization of OSCs.

The authors employed D18-Cl as the polymer donor and BTP-4F-P2EH (Fig. 1a) as the small molecule acceptor to fabricate devices with additive-assisted LBL processing by adding 2% 1-chloronaphthalene (CN) additives to the donor chlorobenzene (CB) solution and 0.5% CN additives to the acceptor chloroform (CF) solution. An excellent gradient fiber morphology was obtained with bulk *p-i-n* structure, significantly decreasing the mixing domain size, promoting exciton dissociation and charge extraction, suppressing trap-assisted recombination and bimolecular recombination, thereby improving the charge generation and transfer process. Besides, the light capture was enhanced by LBL processing facilitated a wrinkle pattern upon deposition of the cathode electrode and reduced photon losses (Fig. 1b). Ultimately, the device based on D18-Cl:BTP-4F-P2EH yielded an unprecedented PCE value of 20.8% (certified by 20.1%), and the device still maintained excellent stability after continuous heating for 400 h and light exposure for 500 h, pushing OSC field to a new era.

**Fig. 1:**
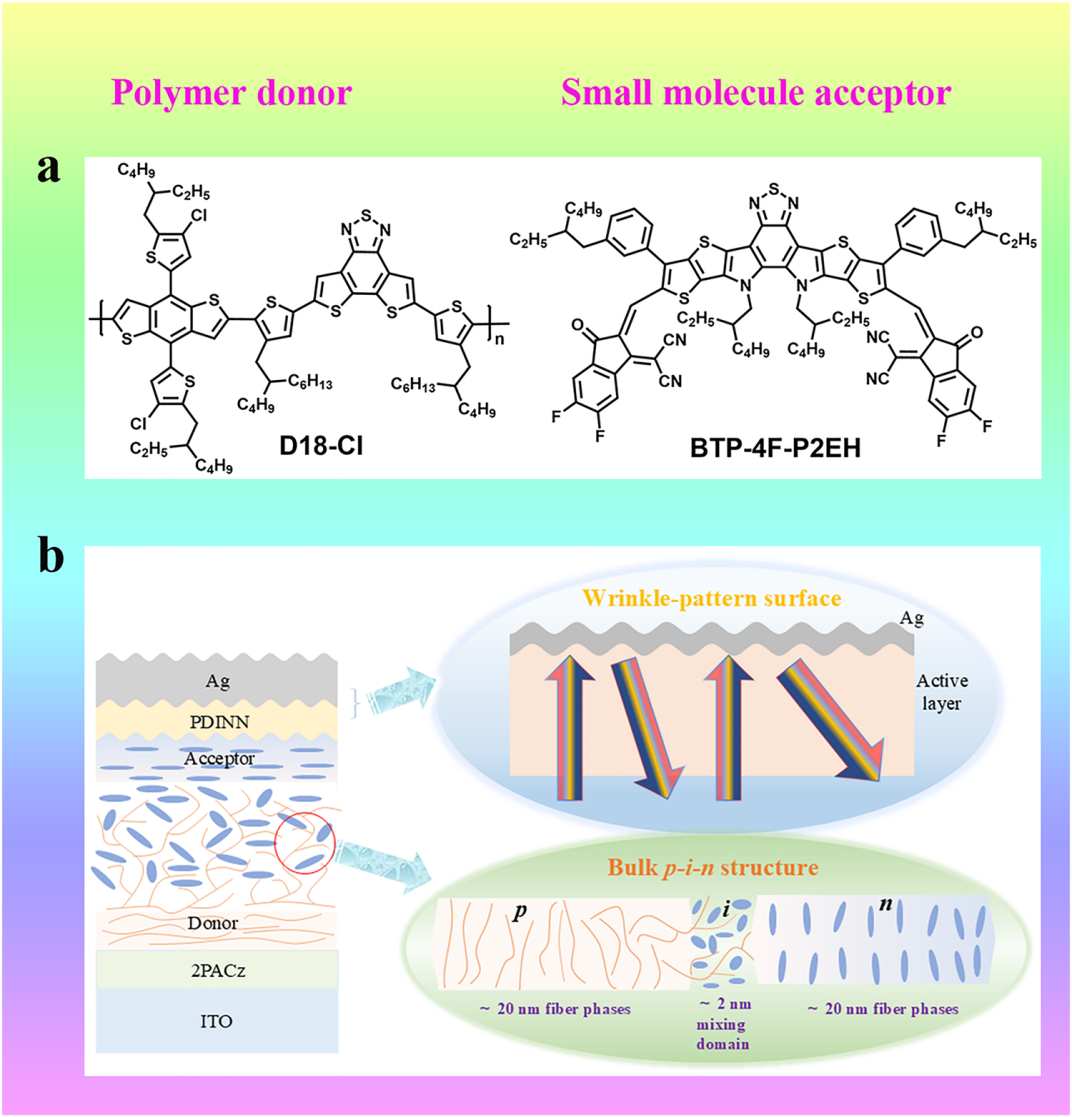
**a** The molecular structures of polymer donor D18-Cl and small molecule acceptor BTP-4F-P2EH. **b** The diagram with bulk p-i-n structure and wrinkle-pattern surface of the D18-Cl:BTP-4F-P2EH-based device

This study holds great significance in the OSC field. Firstly, the efficiency of OSCs is close to 21% up to now, indicating that OSCs may be on the cusp of a new era characterized by an efficiency of 21%, thereby narrowing the gap with the efficiency of other photovoltaic technologies. This development instills confidence and motivation among OSC scientists. Secondly, the micro-structure processed through additive-assisted LBL deposition, which contains about 20 nm fiber phases both the donor and acceptor and about 2 nm *p-i-n* structure, has formed a favorable gradient fiber and 3D morphology, significantly improving device efficiency and stability. This achievement has inspired the OSC community to explore novel methods for precisely controlling morphology and optimizing device performance. Finally, this study underscores the importance of effective light management as another critical factor in achieving higher efficiency and stability in OSCs.

To achieve higher efficiency in OSCs and facilitate their practical commercialization in the near future, it is crucial to emphasize the development of new material systems, the exploitation of new device engeering, and the elucidation of device mechanisms. The general strategies for OSCs include: (1) developing next-generation photovoltaic materials that are both low-cost and high-performance, including donor and acceptor systems; (2) providing a clear interpretation of regulatory morphology, as well as the connections between morphology and efficiency; (3) exploring new device fabrication and optimization techniques and elucidating the mechanisms behind photovoltaic devices in depth; (4) focusing on the commercial development of flexible, semi-transparent, and large-area devices, such as smart integrated photovoltaic windows and wearable electronic products. Researchers are encouraged to tilt toward the direction of market development and strive to solve the key scientific problems in OSC. Ultimately, with the unique advantages and high research value of OSC, we believe that with the efforts of researchers, the spring of OSC market application will surely come.
